# Diagnosis of autism spectrum disorder based on functional brain networks and machine learning

**DOI:** 10.1038/s41598-023-34650-6

**Published:** 2023-05-18

**Authors:** Caroline L. Alves, Thaise G. L. de O. Toutain, Patricia de Carvalho Aguiar, Aruane M. Pineda, Kirstin Roster, Christiane Thielemann, Joel Augusto Moura Porto, Francisco A. Rodrigues

**Affiliations:** 1 Institute of Mathematical and Computer Sciences (ICMC), University of São Paulo (USP), São Paulo, Brazil; 2 BioMEMS Lab, Aschaffenburg University of Applied Sciences, Aschaffenburg, Germany; 3 Health Sciences Institute(HSI), Federal University of Bahia (UFBA), Salvador, Bahia Brazil; 4 Hospital Israelita Albert Einstein, São Paulo, Brazil; 5 Department of Neurology and Neurosurgery, Federal University of São Paulo, São Paulo, Brazil; 6 Institute of Physics of São Carlos (IFSC), University of São Paulo (USP), São Paulo, Brazil

**Keywords:** Computational biology and bioinformatics, Data mining, Data processing, Machine learning, Network topology, Programming language

## Abstract

Autism is a multifaceted neurodevelopmental condition whose accurate diagnosis may be challenging because the associated symptoms and severity vary considerably. The wrong diagnosis can affect families and the educational system, raising the risk of depression, eating disorders, and self-harm. Recently, many works have proposed new methods for the diagnosis of autism based on machine learning and brain data. However, these works focus on only one pairwise statistical metric, ignoring the brain network organization. In this paper, we propose a method for the automatic diagnosis of autism based on functional brain imaging data recorded from 500 subjects, where 242 present autism spectrum disorder considering the regions of interest throughout Bootstrap Analysis of Stable Cluster map. Our method can distinguish the control group from autism spectrum disorder patients with high accuracy. Indeed the best performance provides an AUC near 1.0, which is higher than that found in the literature. We verify that the left ventral posterior cingulate cortex region is less connected to an area in the cerebellum of patients with this neurodevelopment disorder, which agrees with previous studies. The functional brain networks of autism spectrum disorder patients show more segregation, less distribution of information across the network, and less connectivity compared to the control cases. Our workflow provides medical interpretability and can be used on other fMRI and EEG data, including small data sets.

## Introduction

Autism is a multifactorial neurodevelopmental disorder with a complex genetic component^1,2^ and usually manifested since childhood (at least in the first three years of life) through deficits in social communication and restricted, repetitive patterns of behaviors or interests^3^. Because autism spectrum disorder (ASD) varies widely in symptoms and severity, an accurate diagnosis may be difficult. Indeed, there is no medical test to diagnose the disorder, such as a blood test. Diagnosis is based on observing the individual’s communication, social interaction, activities, and interests. This approach depends on experienced professionals, and an incorrect diagnosis can impact families and education, increasing the risk of depression, eating disorders, and self-harm^4^.

Furthermore, an autism misdiagnosis might occur because many other disorders have similar symptoms. In this way, it is essential to develop a quantitative and accurate method for autism diagnosis based on physical exams. This paper considers data from functional brain networks and machine learning algorithms to propose a computer-aid diagnostic methodology for autism.

Our approach is based on previous studies that suggested that autism is a manifestation of changes in the brain organization^5^. Abnormal neuronal connectivity has recently become the essential hypothesis for explaining the symptoms associated with autism^6^. By adopting the fMRI technique, Belmonte and Yurgelun-Todd^7^ demonstrated that the inputs of the autistic brain regions are cut off, with reduced activation and functional correlations with sensory areas. fMRI data from children with ASD^8^ suggest a strong parietal cortex activation responsible for visuospatial and sensory processing. In a resting state, regions of the medial prefrontal cortex related to the executive function comprised of skills that enable the individual to make decisions, pay attention, and differentiate conflicting thoughts are suppressed^9^. Apart from the medial prefrontal region, the rostral anterior cingulate cortex and the posterior cingulate cortex have also been investigated^10^. The function of the former includes memory recall and learning. In contrast, the posterior cingulate cortex is responsible for cognitive, emotional, and learning processes. Its metabolic activities during rest are deactivated during demanding cognitive tasks. According to Kennedy et al.^10^, the midline resting network of patients with ASD is less active than that of the control group, and task deactivation is insignificant. In structural terms, Keller et al.^11^ suggested the development of the brains of autistic children is atypical, showing an early overgrowth of white matter, followed by its reduction in adolescence and adulthood. Furthermore, Diffusion Tensor Imaging (DTI) results revealed the disorganization of white matter paths^12^.

These studies demonstrate that the structure of the brains of autistic people and healthy individuals differ. Therefore, we speculate that autism can be identified by reviewing information on brain anatomical organization. This data can be collected from electroencephalogram (EEG) or functional magnetic resonance imaging (fMRI) experiments. EEG is a relatively inexpensive method readily available in most contexts and has an excellent temporal resolution. Data from EEG has been used to enhance our understanding of human brain structural and functional networks^13–15^. On the other hand, fMRI has a low temporal resolution but a high spatial one, thus being well suited for analyses of spatial brain dynamics^16,17^. fMRI scans produce a set of three-dimensional images recorded over time and measure a signal (called BOLD signal (The decrease in the rate of deoxyhemoglobin can be detected with the increase of the NMR signal. This effect is called Blood Oxygenation Level-Dependent (BOLD))). The temporal evolution of the BOLD series is called the hemodynamic response function and is determined by the pixel intensity in fMRI images^18,19^. Each cube of an fMRI image, called a voxel, which anatomically maps a position in the brain, has a BOLD time series. Here, we consider the BOLD series to develop the classification method for autistic patients.

After mapping the brain, it is possible to classify people with ASD and typical development (TD) using machine learning methods. Machine learning (ML) techniques permit automatically extracting knowledge from a database. Previous studies have evaluated the effectiveness of machine learning in diagnosing ASD with supervised machine learning algorithms that distinguish between two classes, namely ASD and TD. Up to the present date, at least 45 articles have focused on supervised machine learning algorithms that aid in ASD diagnosis, where the most used ones are based on support vector machines (SVM)^20^ (see Table 1 for publications on the use of fMRI for distinguishing between ASD and TD).

**Table 1:** Publications on using supervised ML algorithms on fMRI data for distinguishing ASD from TD patients. Based on^20^.

Authors	Data size	ML methods	AUC	Accuracy	Recall	Precision
^21^	$\hbox {n} = 505$ ASD ASD; $\hbox {n} =530$ TD (ABIDE)	Deep learning	–	0.95	0.97	–
^22^	$\hbox {n} = 462$ ASD; $\hbox {n} =464$ TD (ABIDE)	Deep learning	0.96	0.87	0.87	–
^23^	$\hbox {n} = 126$ ASD; $\hbox {n} = 126$ TD (ABIDE)	RF	0.91	0.89	0.93	–
^24^	(ABIDE)	SVM	–	0.80	–	–
^25^	$\hbox {n} =15$ ASD; $\hbox {n} =45$ TD (ABIDE)	Lasso	0.78	0.75	0.77	0.73
^26^	$\hbox {n} = 505$ ASD; $\hbox {n} =530$ TD (ABIDE)	Deep learning	–	0.70	0.74	0.63
^27^	$\hbox {n} = 505$ ASD; $\hbox {n} =530$ TD	Deep belief network	0.76	–	–	–
^28^	$\hbox {n} = 59$ ASD; $\hbox {n} = 59$ TD	Linear SVM; ridge logistic regression	0.73	–	–	–

Although ML has provided important advances in diagnosing autism, considerable challenges must be addressed. Many classification methods need to be more interpretable, which is disadvantageous, especially for understanding medical data^29,30^. Also, according to Table 1^25,28^, small data sets are quite common^31–34^, which might cause unreliable results. To overcome the lack of interpretability, we can consider new techniques that have emerged in recent years towards facilitating the interpretation of machine learning results (e.g., SHapley Additive ExPlanations (SHAP) values^35^ identify the most important features for a model^36–38^). Moreover, to circumvent the use of small medical data, data augmentation techniques (e.g., sliding windows), which split data (e.g., time series from EEG and fMRI )^39–41^, might be adopted. However, one of their limitations is the loss of information during the splitting process, which the overlapping windows technique can solve. Part of the window information is repeated in each subsequent window and used for EEG^42,43^ and fMRI^44,45^ data. In this paper, we consider these methods to develop a new method for diagnosing autism that is interpretable and can be used in small data sets. In summary, our contributions are the following:

- We design a method to classify fMRI time series using a connectivity matrix as input to the ML algorithm, which provides more accurate results than those reported in the literature.
- Complex network measures characterize brain organization, quantifying the differences between ASD and TD patients. In addition, we use SHAP values for a biological interpretation of the connections between brain regions and their relation with ASD.
- We adopt a sliding window data augmentation approach to increase the sample size by splitting the time series into smaller series with either mutually exclusive sections of the time series or overlapping sections of the sliding windows, in which portions of the sequence are repeated in multiple observations. This approach enables handling small medical data.

It is essential to point out that despite the extensive studies involving ML algorithms for the diagnosis of ASD (as mentioned in Table 1), previous works considered just one pairwise metric, i.e., Pearson correlation^21,22,27^. However, as verified in previous studies (e.g.^46^), correlation metrics are vital for diagnosing mental disorders. Therefore, we considered nine different pairwise metrics to find which best captures the ASD brain changes. Furthermore, unlike the studies in Table 1, we employed the SHAP (SHapley Additive exPlanations) values to identify the connections that differ in ASD and control patients. Moreover, we considered measures of complex networks to analyze how functional brain networks are modified in ASD. Thus, we proposed a more robust methodology that considers not just ML algorithms but also complex network measures while offering a medical interpretation of the results produced.

In the following sections, we describe the dataset, the methodology, and the results.

### Data and data preprocessing

We consider the preprocessed version of the Autism Brain Imaging Data Exchange (ABIDE), which consists of 1112 datasets comprised of 539 ASD and 573 TD with 300s BOLD time series and provided by the Preprocessed Connectomes Project (PCP) dataset^47^. The PCP preprocessing pipeline includes cut time correction, motion correction, intensity normalization, and removal of artifacts such as breathing, heartbeat, and head motion. All data are properly anonymized in compliance with HIPAA requirements, and analyses are conducted following the University of Utah Institutional Review Board’s pre-approved protocols. All images were gathered with informed consent according to procedures established by human subjects research committees at each participating institution. The acquisition, informed consent, and site-specific protocols are described in detail at http://fcon1000.projects.nitrc.org/indi/abide/. Furthermore, it is available for use in Nilearn’s python package, a Python module for neuroimaging data. 242 ASD and 258 TD were used, and the preprocessed data were 0.5 Hz band-pass filtered since recent studies with fMRI have shown fluctuations may exist above that value^48^.

**Figure 1:**
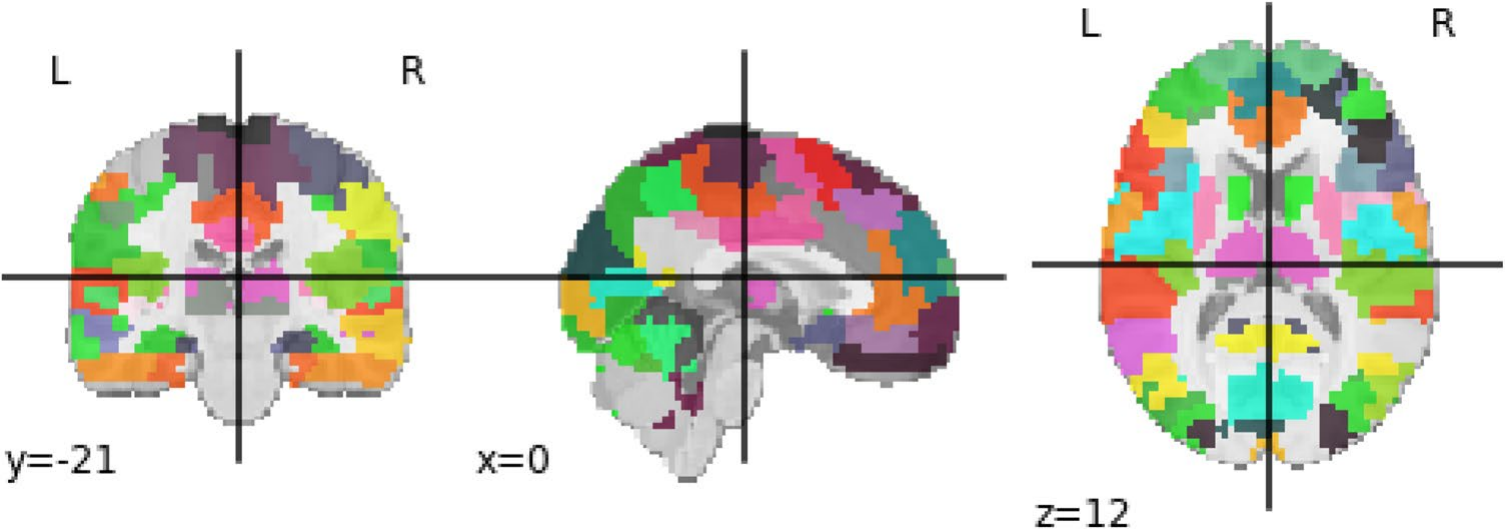
Figure developed using Python package Nilearn and containing BASC atlas with the 122 ROIs used in the present study.

Brain regions of interest (ROI), rather than the entire BOLD time series obtained from each voxel of the brain image, are considered. A brain atlas containing these ROIs is used; therefore, only the BOLD time series voxels of this ROIS were adopted. Among the numerous predefined atlases, Bootstrap Analysis of Stable Clusters (BASC) was chosen since it was the map with the best performance for distinguishing ASD patients by deep learning model, according to^22^. It was proposed in^49^ and generated from group brain parcellation by BASC method, which is a k-means clustering-based algorithm that identifies brain networks with coherent activity in resting-state fMRI^50^. BASC map with a cluster number of 122 ROIs was used here (see Fig. 1). The preprocessed BOLD time series extracted for 122 regions can be found in the Supplementary Information.

A manual use of Yale BioImage Suite Package web application (Avaiable in https://bioimagesuiteweb.github.io/webapp/mni2tal.html) labelled the coordinates of each ROI for the identification of their names. After the extraction of the BOLD time series, the methodology described in “Section Methodology” was adopted.

## Methodology

**Figure 2:**
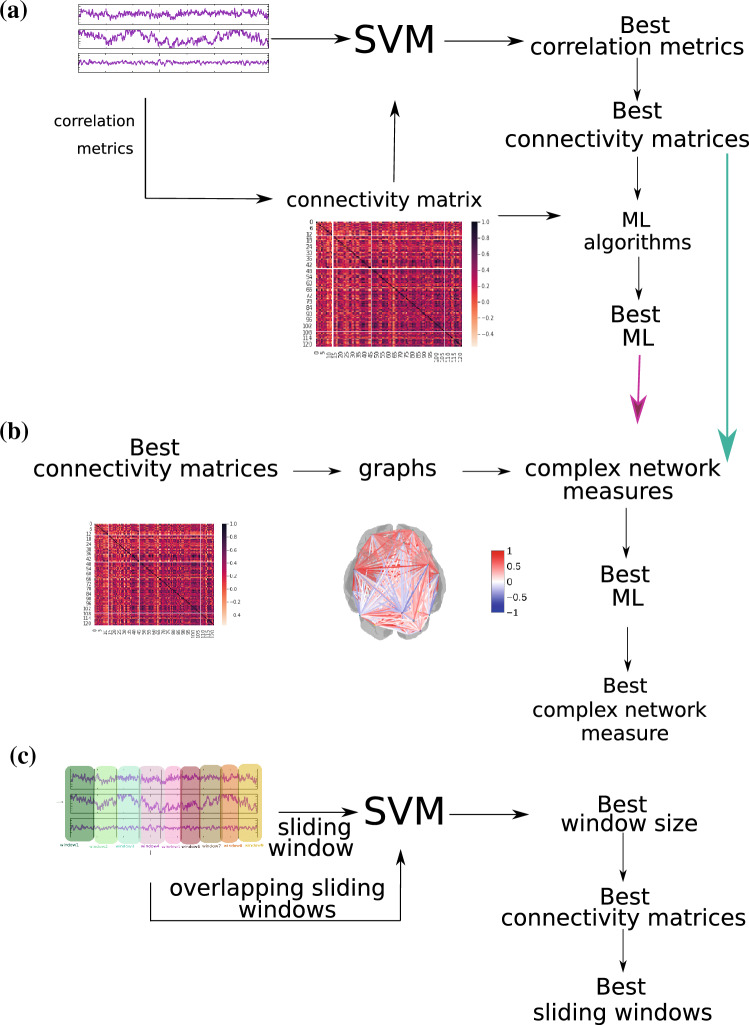
The methodology used here for the diagnosis of autism. (**a**) methodology described in “Section Connectivity matrix”; (**b**) methodology reported in “Section Complex network measures”; (**c**) methodology described in “Section Sliding windows and overlapping sliding windows”.

Figure 2 depicts the methodology workflow used and organized into three parts according to their aim, i.e., the finding of the best connectivity matrix (described in Fig. 2a and in “Section Connectivity matrix”), the best measures of complex networks (described in Fig. 2b and in “Section Complex network measures”), and the best sliding technique for differentiating ASD from TD patients (described in Fig. 2c and in “Section Sliding windows and overlapping sliding windows”). The python code with the methodology used in this work is available at: https://github.com/Carol180619/Paper-autism.git.

### Connectivity matrix

**Figure 3:**
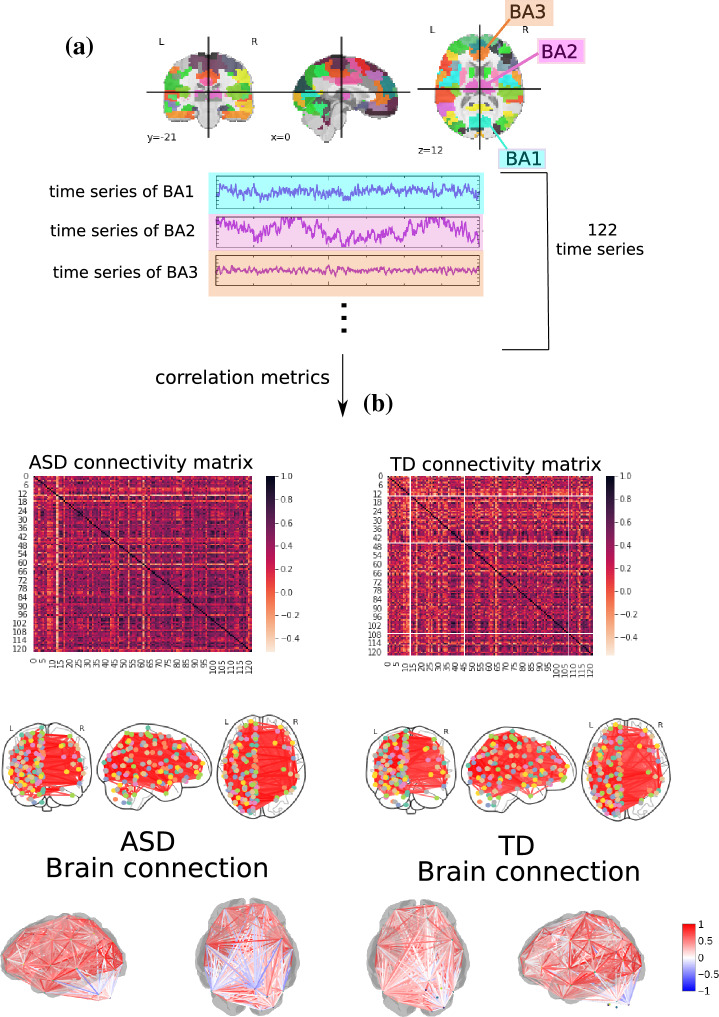
Methodology to obtain the connectivity matrices. In (**a**), time series of 122 ROI is extracted from fMRI data with the use of BASC BOLD atlas (highlighted in blue, purple, and orange). The time series are correlated, (**b**), by pairwise statistical metrics (Pearson correlation was used in this example) towards forming the connectivity matrices, where each row and column correspond to one of the Brodmann areas for a patient with ASD for one with TD. The same highlighted matrices are arranged in a two-dimensional and three-dimensional brain schematic for better visualization.

Once the time series for each of the 122 regions had been extracted, they were correlated according to Pearson Correlation (PC)^51^, Spearman Correlation (SC)^52^, Granger Causality (GC)^53^, Biweight Midcorrelation (BM)^54^, Sparce Canonical Correlation analysis (SCC)^55^, Graphical Lasso method (GL)^56^, Ledoit-Wolf shrinkage (LW)^57^, Mutual Information (MI)^58^, and Transfer Entropy (For the TE, MI, and GL metrics, a Min-max normalization and then a thresholding process was performed, with a value of 0.5, since these measures deal best with binary values) (TE)^59^. Finally, Fig. 3 displays the scheme to generate the connectivity matrices.

Each matrix was reduced to the size of the vectors used as input to the ML algorithm. The support vector machine (SVM) algorithm^60^ was used to select the best methods to construct the correlation and connectivity matrices. We use this method because it has been considered in studies of ASD (see “Section Introduction”) and has a lower computational cost. The time series of each ROI was used for directly feeding SVM and finding the best connectivity metric that captured the brain changes due to ASD. It also checked whether the use of metrics was better than the direct use of time series - the one of better performance would be chosen. The results can be found in “Section Results related to the pairwise metrics”.

After the best brain connectivity metric had been determined, the following ML classifiers were used: Random Forest (RF)^61^, Naive Bayes (NB)^62^, Logistic regression (LG)^63^ with L-BFGS (Limited-memory Broyden Fletcher Goldfarb Shanno) solver^64^, Multilayer Perceptron (MLP)^65^, and tuned convolution neural network (called here tuned CNN) implemented in^46^. The SHAP value method was used for biological interpretation since it explains individual predictions of each attribute. The same sampling data set was used in all ML algorithms and split into training (train) and test sets, with $25\%$ of data comprising the test set. A k-fold cross-validation procedure was employed, with k = 10—this is a very used value for this method^66–70^). This procedure is used for model selection and hyper-parameter optimization. We considered the method called grid search, which was used for all ML algorithms except the untuned CNN model (since deep learning algorithms have a higher computational cost), as done in^71–75^. The hyper-parameter optimization values for each classifier model are provided in the “Appendix”. The standard performance metric accuracy^76–80^ was employed for evaluation. Due to the two-class (negative and positive) classification problem, other common metrics such as precision and recall were considered^81–84^. Precision (also called positive predictive value) corresponds to the hit rate in the negative class (here corresponding to the TD group), whereas recall (also called sensitivity) measures how well a classifier can predict positive examples (hit rate in the positive class), here related to ASD patients. F1 score^72,85,86^, another well-known measure, is the harmonic mean of recall and precision^87^. Regarding the visualization of the two latter measures, the Receiver Operating Characteristic (ROC) curve is a common method that displays the relation between the rate of true and false positives. The area below the curve, called Area Under ROC Curve (AUC), has been widely used in classification problems^74,76,88,89^. The AUC value ranges from 0 to 1- 1 corresponds to a classification result free of errors, and 0.5 indicates the classifier cannot distinguish the classes, as in a random choice. The micro average of the ROC curve, which computes the AUC metric independently for each class (it calculates AUC for healthy individuals, class zero, and separately calculates it for unhealthy ones, class one), was also considered. The average is computed considering the classes equally. The macro average was also employed in our evaluation - it does not consider the classes equally but aggregates their contributions separately and then calculates the average. The ML algorithms results can be found in “Section ML algorithms results”.

### Complex network measures

A complex network (or a graph) was generated for each connectivity matrix to extract different measures. Towards inputting data into the ML algorithm, the complex network measures were stored in a matrix of attributes. Each column represents a complex network measure (feature), and each row denotes a subject. 2D matrices were generated for all subjects, as in^90^.

To describe the brain structure, the following complex network measures were calculated: assortativity coefficient^91,92^, betweenness centrality (BC)^93^, average shortest path length (APL)^94^, closeness centrality (CC)^95^, diameter^96^, hub score^97^, average degree of nearest neighbors^98^ (Knn), eigenvector centrality (EC)^99^, mean degree^100^, second moment of the degree distribution (SMD)^101^, entropy of the degree distributuion (ED)^102^, transitivity^103,104^, complexity, k-core^105,106^, eccentricity^107^, density^108^, and efficiency^109^.

Newly developed metrics (described in detail in^90^) reflecting the number of communities in a complex network were also applied. Community detection algorithms were also used in our study^110–112^. Since the community detection measures must be transformed into a single scalar value to be included in the matrix, community detection algorithms were applied to find the largest community. The average path length within the community was then calculated and received a single value as a result. The community detection algorithms used were the fastgreedy (FC)^113^, Infomap (IC)^114^, leading eigenvector (LC)^115^, label propagation (LPC)^116^, edge betweenness (EBC)^117^, spinglass (SPC)^118^, and multilevel community identification (MC)^119^. The abbreviations were extended with the letter “A” (for average path length) to indicate the approach (AFC, AIC, ALC, ALPC, AEBC, ASPC, and AMC).

These network measures were utilized to characterize the brain structure. Thus each observation (which represents the Patient’s brain network) is represented by a vector with these metrics. The results are provided in “Section Results for complex networks measures”.

### Sliding windows and overlapping sliding windows

Due to the common issue of small datasets in neuroscience, the previously described methodology was expanded by a sliding window data augmentation approach. First, the sample size was increased by splitting each time series into smaller series. Such an increase can be achieved with either mutually exclusive sections of the time series or overlapping sections of the sliding windows, in which portions of the sequence are repeated in multiple observations.

A sample with 50 patients (25 ASD and 25 TD) was considered from the initial sample (242 ASD and 258 TD) for window sliding and overlapping windows sliding techniques evaluations. Then, the BOLD time series with 300 s were divided into windows of 15, 20, 30, 50, and 60 s and placed into the SVM to check the best way to split data. Then, with the best window size, it was also considered overlap sizes of 10%, 15%, 25%, 35%, 45%, and 55% of it. In other words, if the overlapping is 10%, each sliding window size has depicted a repetition of 10% of the previous window. This approach is used to avoid losing information when sliding.

The connection matrices are constructed using the best partitioning technique and the best correlation metric that fed the previously computed best classifier (see Fig. 2). The same sliding workflow was considered with samplings of 10, 20, 30, 50, 124, and 188 patients. The choice of such different sizes was based on previous neuroscience studies that used fMRI of similar sample sizes, respectively^120–125^.

Additionally to the performance metrics, a mean square error (MSE) was obtained for each sampling and each iteration of the k-fold cross-validation, resulting in an error vector. It was compared with the vector of the MSE obtained using the whole sample by statistical Student’s paired t-test^126^. The results are provided in “Section 3.4”.

## Results

ML algorithms were applied for three different levels of data abstraction, namely (A) the connectivity matrix, (B) the matrix of attributes, whose elements are complex network measures calculated from (A), and (C) sliding data (see Fig. 2). In addition, the sliding window method was employed as an augmentation technique on small data samples to evaluate whether this methodology is advantageous when dealing with these data sets. We verify that all approaches automatically detected changes in the brain of ASD patients. The highest classification performance was obtained for the connectivity matrix with a 99% mean AUC (Table 2). Sections “Results related to the pairwise metrics”,  “Results for complex networks measures”, and “Results from sliding windows and overlapping sliding windows” detail the results.

**Table 2:** The table contains the summary of all results of the present work.

Data abstraction level	Subset	AUC	Accuracy	F1	Recall	Precision
Connectivity matrix	Train	**1.00**	**1.00**	**1.00**	**1.00**	**1.00**
	Test	**0.99**	**0.99**	**0.99**	**0.99**	**0.99**
Complex network	Train	0.93	0.93	0.89	0.88	0.91
	Test	0.98	0.98	0.98	0.98	0.98
Sliding data	Train	0.84	0.84	0.84	0.84	0.84
	Test	0.81	0.81	0.81	0.81	0.81

Classification using the data abstraction connectivity matrix best-captured brain changes due to ASD. The best performance is highlighted in bold.

### Results related to the pairwise metrics

Table 3 contains the results for each connectivity matrix with different types of pairwise statistical metrics. SVM was used to detect the best one for capturing the brain changes due to ASD.

**Table 3:** Results from different ML algorithms.

Measures	Subset	AUC	Accuracy	F1	Recall	Precision
Time series	Train	0.49	0.51	0.00	0.27	0.35
	Test	0.50	0.51	0.34	0.50	0.26
PC	Train	0.67	0.67	0.69	0.67	0.66
	Test	0.60	0.60	0.60	0.60	0.60
SC	Train	**0.98**	**0.98**	**0.97**	**0.98**	**0.98**
	Test	**0.98**	**0.98**	**0.98**	**0.98**	**0.98**
GC	Train	0.51	0.52	0.00	0.32	0.37
	Test	0.50	0.51	0.34	0.50	0.26
BM	Train	0.75	0.75	0.72	0.75	0.75
	Test	0.75	0.75	0.75	0.75	0.75
SCC	Train	0.67	0.67	0.65	0.67	0.66
	Test	0.62	0.62	0.62	0.62	0.62
GL	Train	0.66	0.66	0.65	0.66	0.66
	Test	0.57	0.58	0.57	0.57	0.57
LW	Train	0.66	0.66	0.64	0.66	0.65
	Test	0.58	0.58	0.58	0.58	0.58
MI	Train	0.49	0.50	0.40	0.50	0.49
	Test	0.49	0.49	0.49	0.49	0.49
TE	Train	0.90	0.90	0.89	0.90	0.90
	Test	0.91	0.91	0.91	0.91	0.91

The best MLs were RF and LR, whose performances are highlighted.

Significant values are in bold.

Spearman correlation coefficient (SC) achieved the best performance, followed by transfer entropy (TE). Finally, the best connectivity matrix was tested with the other ML algorithms to determine which best differentiated ASD patients from TD ones.

### ML algorithms results

**Figure 4:**
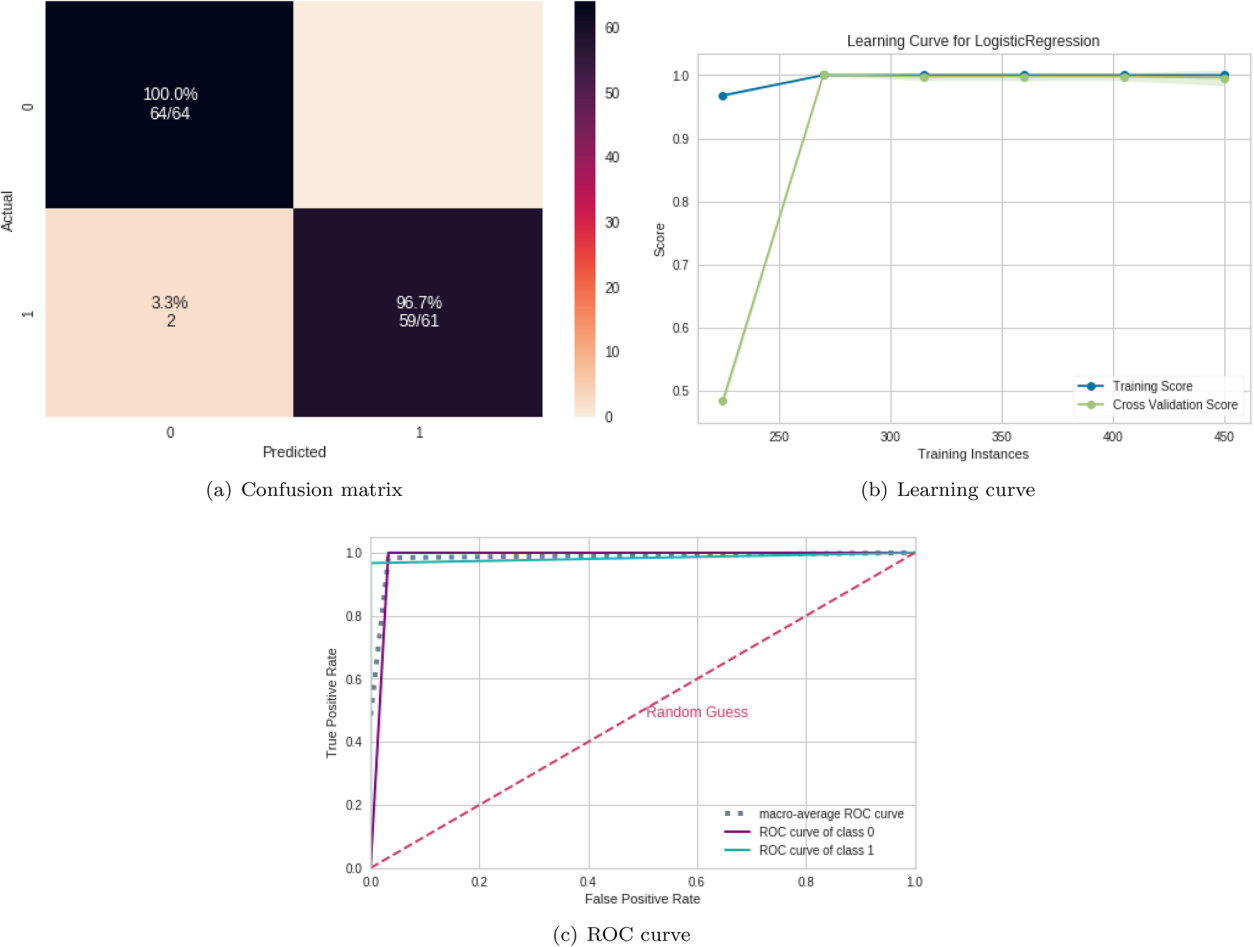
ML results from connectivity matrices. (**a**) Confusion matrix indicating a $96.7\%$ TN rate (purple, according to the color bar) and a $100\%$ TP rate (blue, according to the color bar). (**b**) The learning curve for the training accuracy (blue) and for test accuracy (green). (**c**) ROC curve with classes 0 (TD) and 1 (ASD).

According to Table 4, the best classifiers are the random forest (RF) and logistic regression (LR). Since LR has a lower computational cost, it was chosen for the next steps. Its performance for the test set was equal to 0.99 for the mean AUC, precision, F1, recall, and accuracy. Figure 4 displays the confusion matrix (Fig. 4a), the learning curve (Fig. 4b), and the ROC curve (Fig. 4c), respectively.

The learning curve evaluates the model’s predictability by varying the size of the training set^38^. The results show that the entire database is optional for achieving the highest validation accuracy. Regarding the classification model, TP (related to class 1) was higher than TN, showing that it better detects ASD patients (see confusion matrix in Fig. 4b).

**Table 4:** Results from different ML algorithms.

ML algorithm	Subset	AUC	Accuracy	F1	Rec.	Pre.
SVM	Train	0.99	0.99	0.99	0.99	0.99
	Test	0.98	0.98	0.98	0.98	0.98
RF	Train	**1.00**	**1.00**	**1.00**	**1.00**	**1.00**
	Test	**0.99**	**0.99**	**0.99**	**0.99**	**0.99**
NB	Train	1.00	1.00	0.99	1.00	0.99
	Test	0.98	0.98	0.98	0.98	0.98
LR	Train	**1.00**	**1.00**	**1.00**	**1.00**	**1.00**
	Test	**0.99**	**0.99**	**0.99**	**0.99**	**0.99**
MLP	Train	1.00	1.00	1.00	1.00	1.00
	Test	0.98	0.99	0.99	0.99	0.99
Untuned CNN	Train	1.00	1.00	1.00	1.00	1.00
	Test	0.86	0.87	0.92	0.94	0.90

The best ML was RF and LR, whose performances are highlighted.

Significant values are in bold.

**Figure 5:**
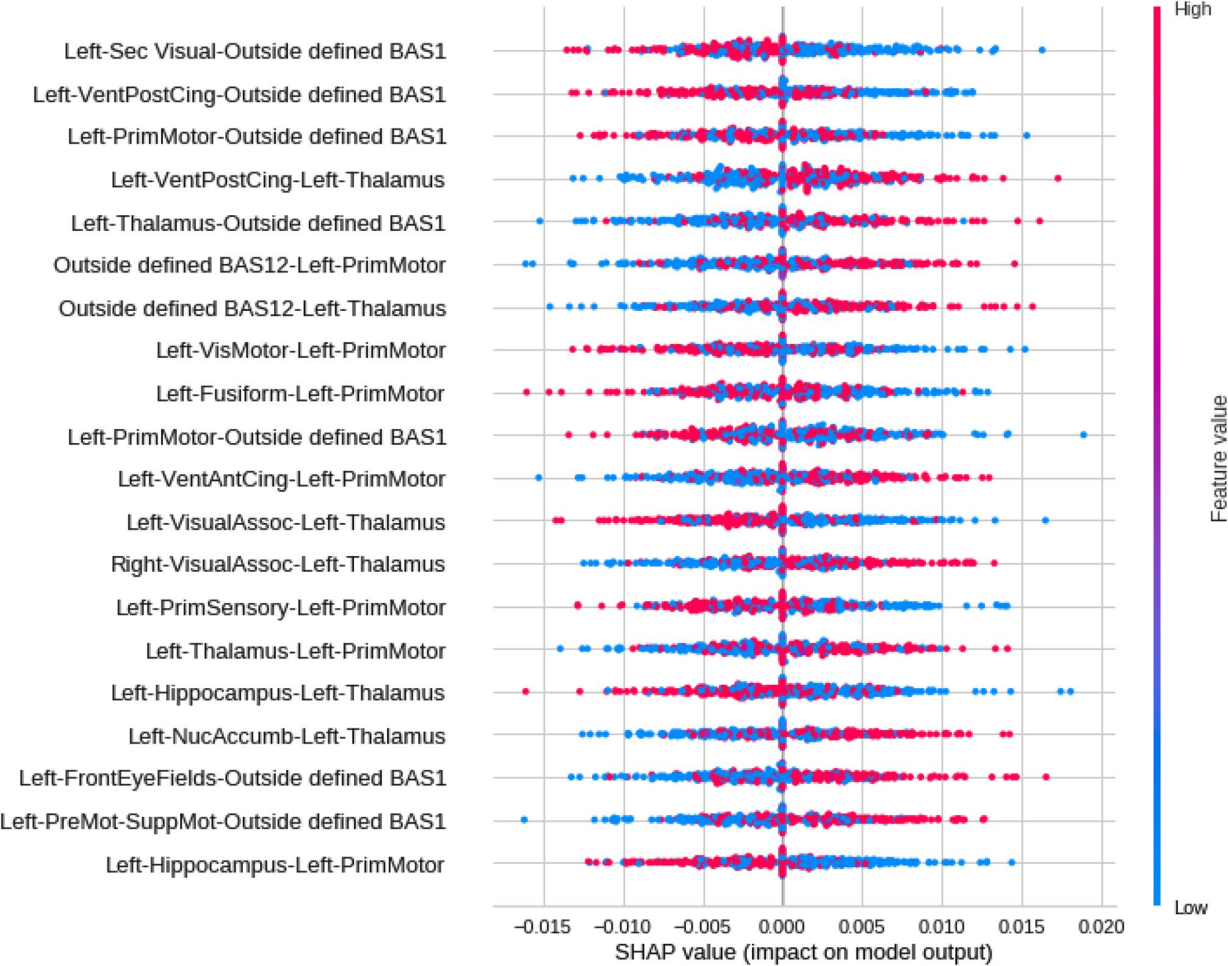
Feature importance ranking for the LR classifier with brain regions in descending order. The connection between the Left-Sec Visual and Outside defined BAS1 regions is the most important for classifying ASD patients.

**Figure 6:**
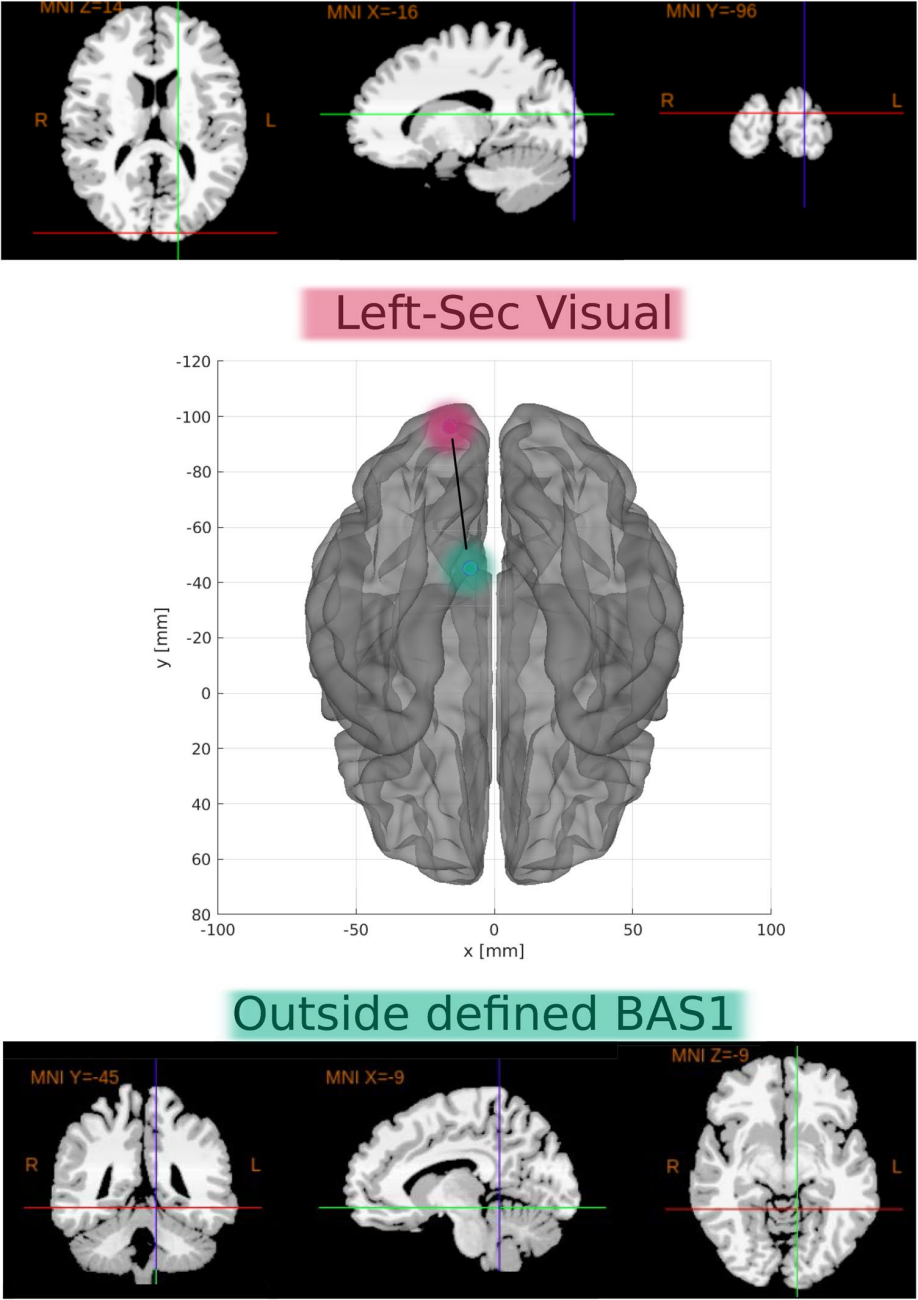
The most important connection found. Two-dimensional schematic (ventral-axis), where the connection between the Left-Sec Visual region (visual cortex, highlighted in pink) and Outsides BAS1 (cerebellum, highlighted in green) is observed in the central region. The brain plot was developed by the Braph tool^127^, and each region was plotted using the Brodmann map from Yale BioImage Suite Package (upper and lower regions in the Figure).

SHAP values were calculated to quantify the importance of brain connections for the logistic regression classifier (LR) (see Fig. 5 for the results). The area between regions Left-Sec Visual (visual cortex) and Outside defined BAS1 (area outside Brodmann’s map), identified as the cerebellum, was the most important connection. According to the data in Fig. 5, low correlation values (blue dots) for the connection (Left-Sec Visual and Outside defined BAS1) were essential for the detection of ASD patients, and high values of correlation (red dots) were important for the detection of TD ones. The second most crucial connection was detected between the Left ventral posterior cingulate cortex (Left-VentPostCing) and, again, the cerebellum (Outside defined BAS1). Figure 6 depicts the corresponding brain regions.

**Figure 7:**
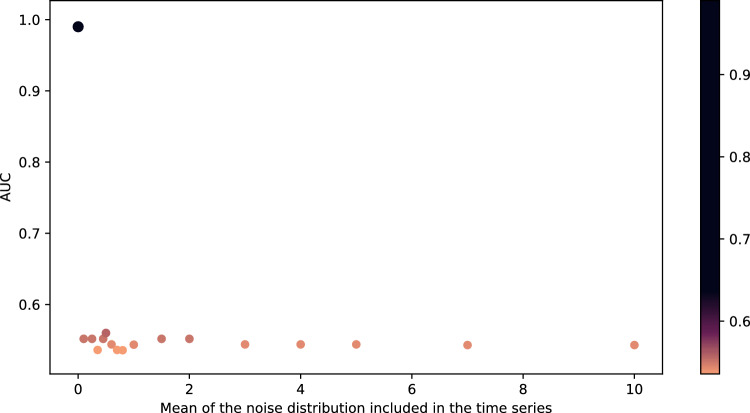
The mean AUC of the test was obtained with the insertion of noise generated by a normal distribution with 0.1 standard deviation and 0–10 mean range.

Since LG was the algorithm that provided the best performance, it was used in the following subsections. Furthermore, since the results were close to 100%, noises were inserted into the ASD and TD time series for further testing the model in this study. Such noises were generated by a normal distribution with a standard deviation equal to 0.1 and mean on the interval [0, 10]. After introducing the noises, Spearman’s correlation was used to generate the connectivity matrices from the time series. The results of the average AUC calculated on the test set are shown in Fig. 7. According to Fig. 7, the AUC according to the noise follows approximately a decreasing logarithmic function.

### Results for complex networks measures

**Figure 8:**
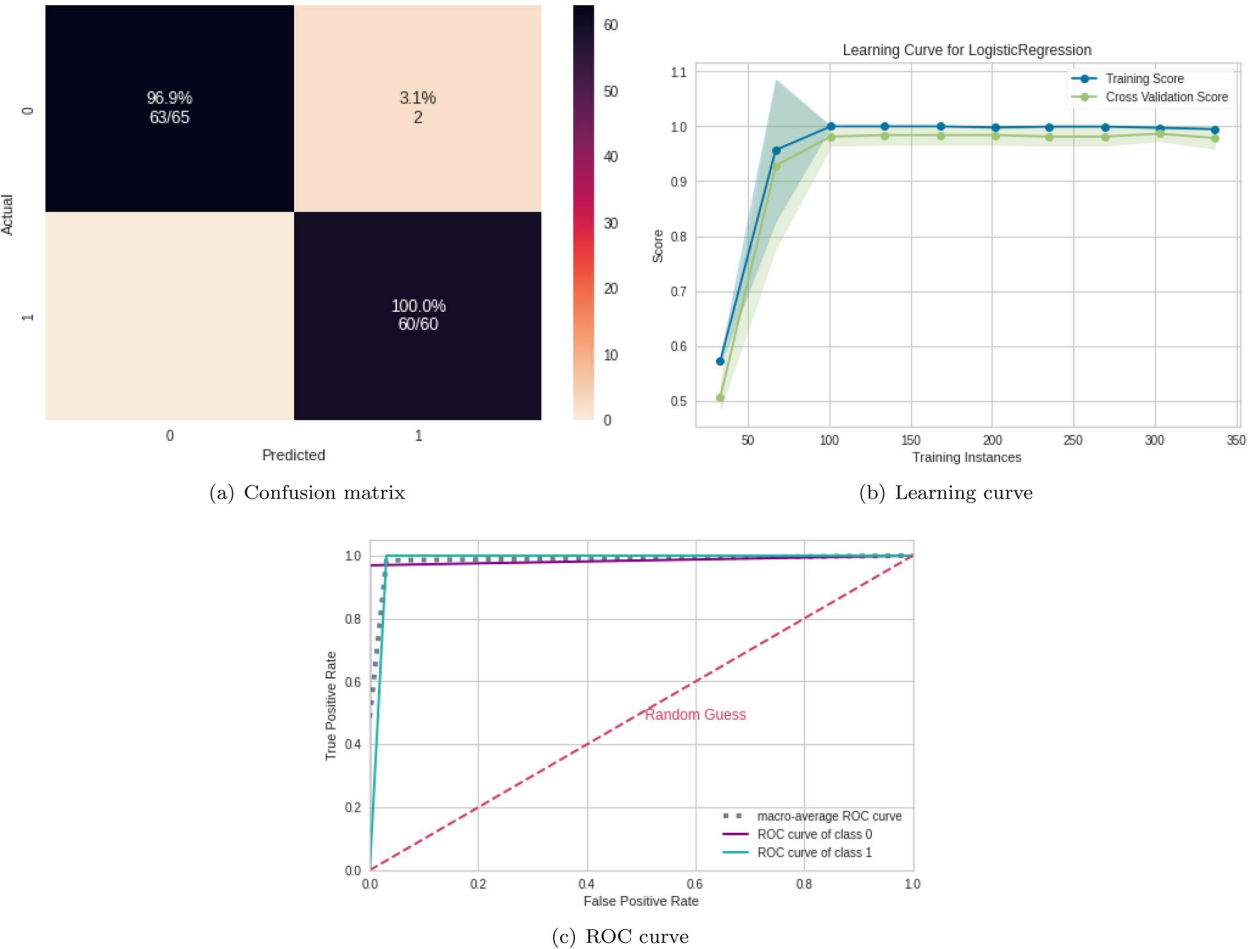
ML results from complex network measures. (**a**) Confusion matrix indicating a $98.5\%$ TN rate (blue, according to the color bar) and a $98.3\%$ TP rate (purple, according to the color bar). (**b**) The learning curve for the training accuracy (blue) and for test accuracy (green). (**c**) ROC curve with classes 0 (TD) and 1 (ASD).

The performance of the test sample considering the complex network yields a mean AUC equal to 0.98, 0.98 for precision, 0.98 for F1 score, 0.98 for recall, and 0.99 for accuracy. Confusion matrix Fig. 8, learning curve Fig. 8, and ROC curve Fig. 8 are shown in Fig. 8. Furthermore, according to Fig. 8, the whole dataset was unnecessary because the best result could be reached with only 100 train instances.

**Figure 9:**
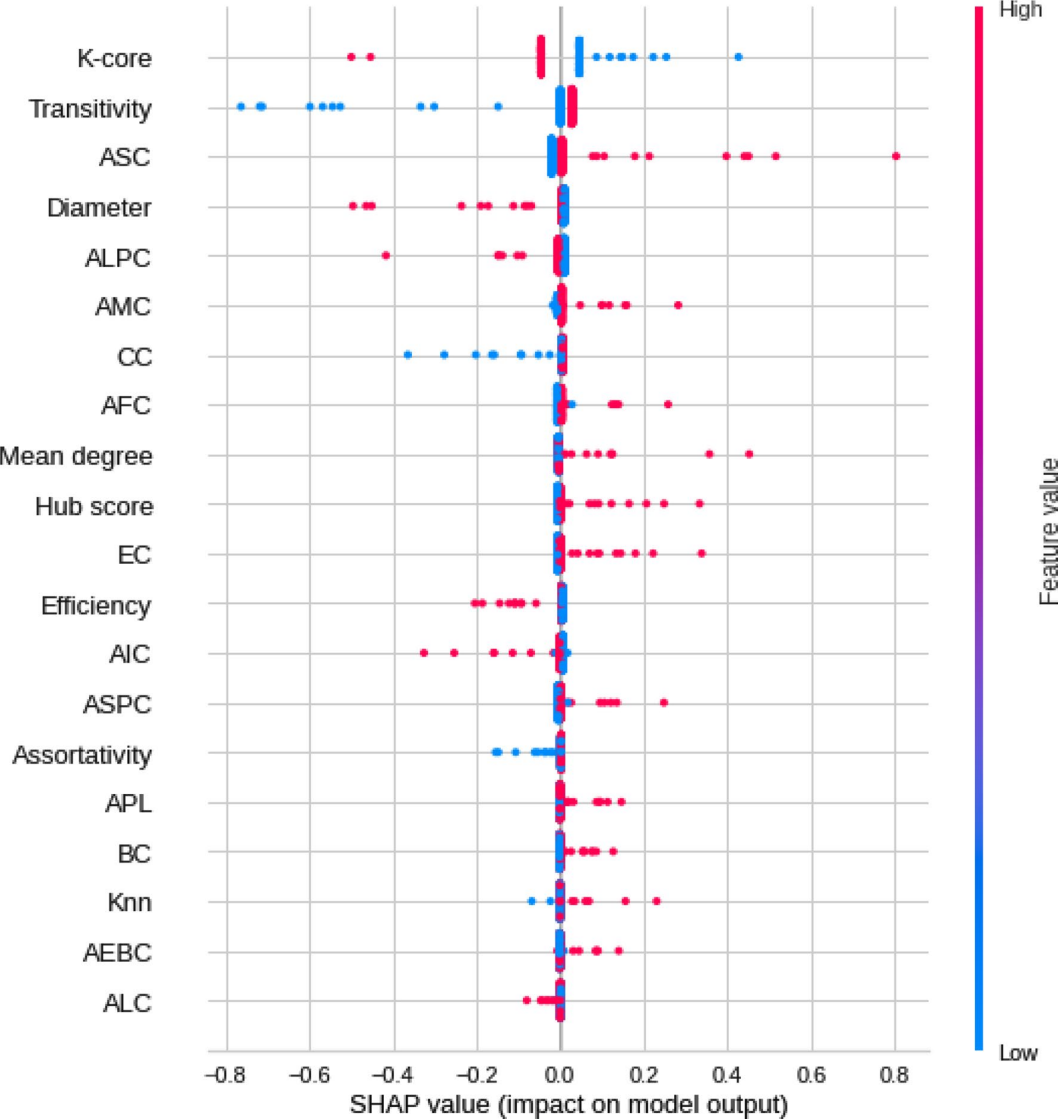
Feature importance ranking for LR classifier with features in a descending order. K-core measure is the most important for the classification of ASD patients, followed by the AEBC measure.

According to the SHAP values in Fig. 9, the most crucial measure for the model was the k-core, followed by the AEBC, introduced in^90^. High k-core values (pink dots) indicate their importance for the detection of TD, and low ones (blue dots) are important for the detection of ASD (Fig. 9). Low AEBC values (blue dots) indicate its importance for the detection of ASD, and high ones (pink dots) suggest its importance for the detection of TD. Higher values of efficiency were associated with TD patients; higher values of transitivity were associated with ASD, and low values indicated TD. Remarkably, the seven measures introduced in^90^ appeared in the ranking of best ones.

### Results from sliding windows and overlapping sliding windows

**Figure 10:**
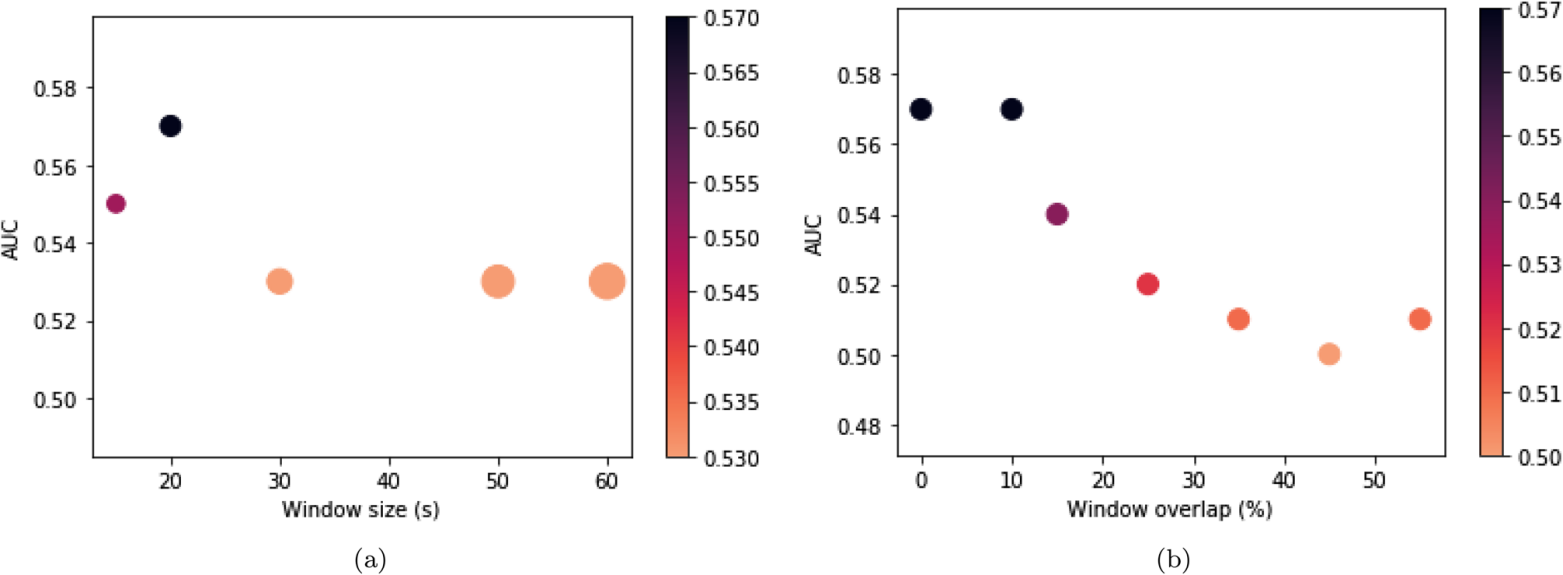
Results of sliding and overlapping window sizes. (**a**) Mean AUC test obtained for the different window sizes. The width of the points in the graph corresponds to the window size variation, and their colors are indicated in the color bar. (**b**) The mean AUC test was obtained for the different overlapping sizes corresponding to the percentage of the window size. The colors of the points in the graph are depicted in the color bar.

In this section, since two data augmentation techniques have been considered, a sample with 50 patients (25 ASD and 25 TD) was considered from the initial sample (242 ASD and 258 TD). Figure 10a shows the performance of SVM fed by time series divided into different window sizes. The best performance was achieved with a window size of 20 s. Figure 10b shows the best performance obtained with no overlapping or with a 10% of the time window size. Consequently, 10% overlapping was considered for the next step to avoid loss of information in the sliding process.

The sliding process was used with different sample sizes, and the results are shown in Table 5.

Paired Student’s t-test (here called t-test) was also calculated between the sample performance and the performance for the whole data set. The null hypothesis is that the performances were statistically different. Therefore, a sample size of only ten patients was taken as a basis for comparison, given the premise that their performance should be statistically different when the entire database is considered for such a small sample size. Only samples for which the null hypothesis could be rejected (p-value greater than or equal to the baseline value for comparison) were considered, i.e., 10 and 20 patients. In other words, the performance of those two sizes showed no statistically significant differences from the data set but very similar results (Table 5). In other words, the performance of these two sample sizes showed no statistically significant differences from the data set but very similar results.

**Table 5:** Performance of the LR algorithm with the use of the sliding process and a varied number of samples of TD and ASC patients.

Data abstraction level	Subset	AUC	Acccuracy	F1 score	Recall	Precision	T-test
Sample 10	Train	0.79	0.80	0.80	0.77	0.81	7.25 e-07 Threshold p-value
	Test	0.80	0.80	0.80	0.80	0.80	
Sample 20	Train	0.80	0.80	0.80	0.77	0.80	7.11 e-05 Reject null hypothesis
	Test	0.80	0.80	0.80	0.80	0.80	
Sample 30	Train	**0.84**	**0.84**	**0.84**	**0.84**	**0.84**	**3.14 e-06 Reject null hypothesis**
	Test	**0.81**	**0.81**	**0.81**	**0.81**	**0.81**	
Sample 50	Train	0.75	0.75	0.75	0.76	0.75	1.24 e-08
	Test	0.75	0.75	0.75	0.75	0.75	
Sample 124	Train	0.70	0.70	0.70	0.72	0.70	2.77e-09
	Test	0.73	0.73	0.73	0.73	0.73	
Sample 188	Train	0.70	0.70	0.70	0.70	0.70	7.60 e-12
	Test	0.74	0.74	0.74	0.73	0.74	

The best performance is highlighted in bold and was achieved with 30 patients.

**Figure 11:**
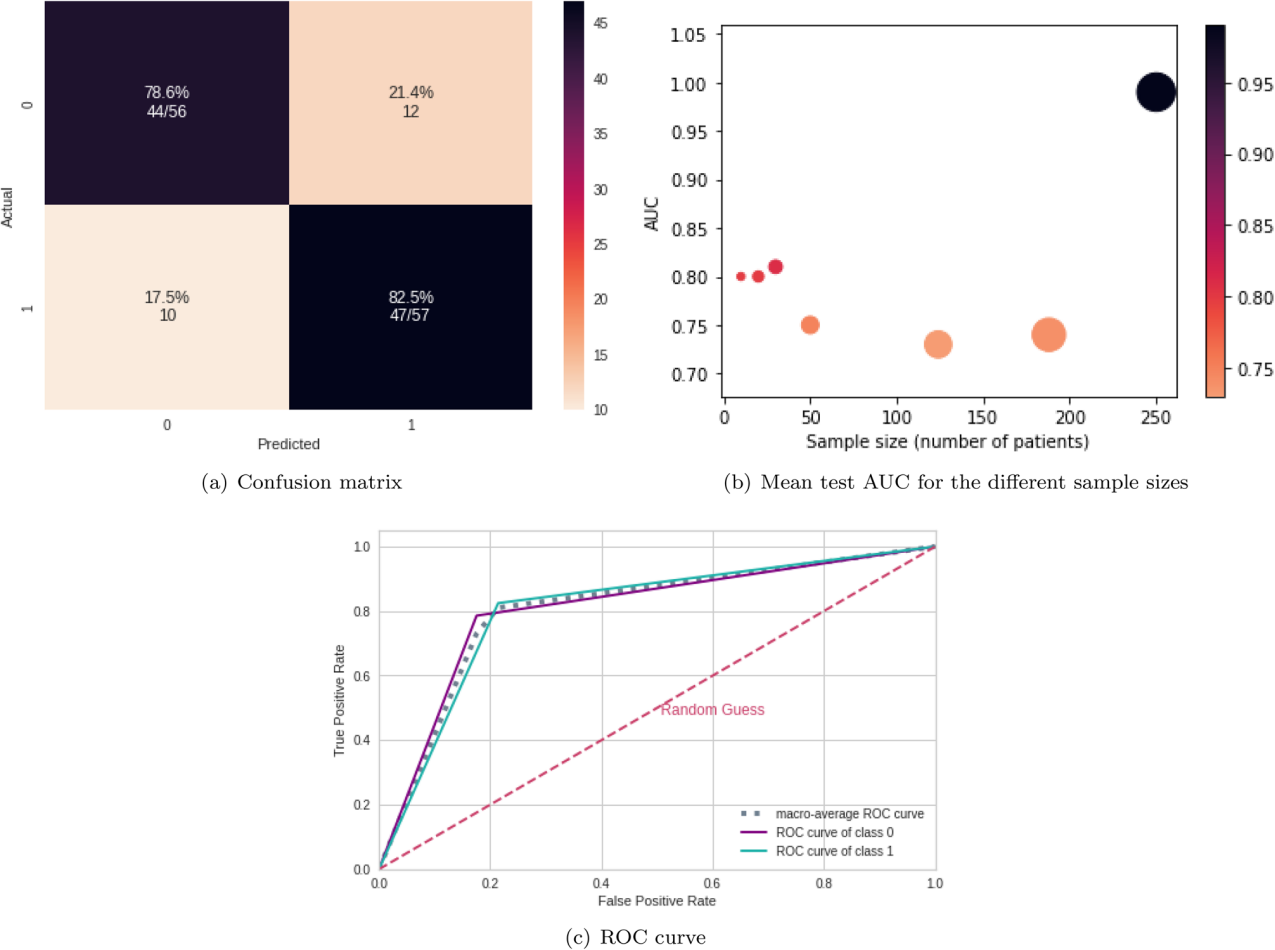
Results from connectivity matrices and ML. (**a**) Confusion matrix indicating an $82.5\%$ true negative rate (blue, according to the color bar) and a $78.6 \%$ true positive rate (purple, according to the color bar). (**b**) Mean test AUC was obtained for the different sample sizes; the width of the points in the graph corresponds to the window size variation, and their colors are depicted in the color bar. (**c**) ROC curve with classes 0 (TD) and 1(ASD).

Figure 11 shows the confusion matrix (Fig. 11a) for the sample size of 30 patients, the mean AUC test for each sample size (Fig. 11b), and the ROC curve for the sample size of 30 patients (Fig. 11c). According to Fig. 11b, ASD and TD patients were differentiated even with different sample sizes, with above 79% AUC and accuracy.

## Discussion

The results from using the abstraction levels of the connectivity matrix and complex network data were superior to those reported in the literature (see Table 1). Therefore, the workflow developed here is more effective for detecting ASD patients with above 95% mean accuracy and mean AUC, and SC was the measure that best-captured brain changes in the patients (as an example, it is more robust for non-linear correlations than PC^128^). Since the Pearson correlation coefficient (PC) was ineffective in discriminating between the two classes (ASD and control subjects), we can conclude that brain changes due to ASD have a non-linear nature. Also, LG provides the best results, being the most suitable machine learning model, with lower computational cost than other ML algorithms used here (such as untuned CNN). Furthermore, we obtained better precision and recall compared with the studies presented in Table 1. A higher precision indicates that our model can better infer that an element belongs to class one (with ASD). In contrast, a higher recall implies that more elements with class one (with ASD) are captured. Furthermore, we obtained better precision and recall compared with the studies presented in Table 1. A higher precision indicates that our model can better infer that an element belongs to class one (with ASD). In contrast, a higher recall measure indicates very few false negatives, in our case, elements from class one, with ASD, and the model classifies as TD, which is helpful for medical data for correct diagnosing.

The most important connection in the first five significant correlations was observed between Left-Sec Visual (visual cortex) and cerebellum (Outside defined BAS1) regions. Low correlation values (blue dots) were important for detecting ASD patients, whereas high values (red dots) indicated TD. The second most crucial connection was established between the Left- VentPostCing and, again, the cerebellum (Outside de- fined BAS1) regions. Finally, the cerebellum (Outside defined BAS1), Left-Thalamus, and Left-Prim Motor appeared in several primary connections. Notably, Left-Thalamus has been reported in other studies associated with ASD^129,130^.

Left-Sec Visual (visual cortex) is a part of the cerebral cortex that processes visual information, and a lower connection to the cerebellum (Outside defined BAS1) is more associated with ASD.

Left-VentPostCing corresponds to the upper part of the limbic system, i.e., part of the brain involved in behavioral and emotional responses. According to the literature, reductions in the functional connectivity of that brain area are expected in ASD patients^131^, which is consistent with our results since the region is less connected to the Outside BAS1 in ASD patients.

The brain region changes addressed elsewhere have been reported in the ASD literature. For example, both hyper- and hypo-connectivity were observed in ASD through stepwise functional connectivity in the resting state^132^. In the same study, hypoconnectivity was related to the parietal and frontal regions of the attention networks, whereas hyperconnectivity was observed for the default mode network in the visual cortex region. The authors in^133^ claimed ASD patients have higher activity in the occipital cortex bilaterally and in the Anterior Cingulate Cortex (ACC) but lower activation in the frontal gyri in comparison with a control group during automatic identification of visual changes. However^131^, reported reduced functional connectivity in ACC in ASD patients. The low correlation observed between the posterior cingulate region and the cerebellum (Left-VentPostCing vs. Outside BAS1) found in our study seems to point to a dysfunction, i.e., an alteration in functional communication in ASD. Such a correlation differs from the findings for ASD reported by other researchers, who have pointed to the anterior cingulate as one of the altered brain regions in ASD^131,133,134^ and found cortical thinning for ASD in the right ACC. Such results have led us to hypothesize that the ACC and other cingulate regions are implicated in ASD. Moreover, our attention has been drawn to the cingulate region and its relationships with other brain regions. The hypothesis can be reinforced by the findings of^135^, who reported abnormal functional connectivity between the posterior cingulate cortex and the ventromedial prefrontal cortex for ASD, with hypoconnectivity. Other studies have shown ASD patients have altered intra- and inter-network connectivity among the cerebellum, visual networks, and the sensory-motor region. According to^136^, the connectivity among those regions is related to problems in sensory and visual motor integration present in ASD. Such findings have corroborated our results of a low correlation between visual cortex regions and the cerebellum (first correlation of highest importance) and a correlation between the left primary motor region and the cerebellum (third correlation of highest importance). The cerebellum is associated with motor functions such as balance maintenance, executive control of movements, and cognitive, behavioral, and language functions^137–141^. Studies with fMRI have pointed to structural and functional changes in several cerebellum regions related to ASD. Lesions in the cerebellum compromise the cognitive, perceptual, and motor functioning of those systems^142^. Stoodley^143^ claimed abnormalities in the different cerebellar regions would produce behavioral symptoms associated with the functional breakdown of specific cerebrocerebellar circuits, thus compromising the acquisition of certain skills. Moreover, such long-term changes would significantly impact behavior, language, and social cognition, hence dysfunctions in behaviors associated with ASD, dyslexia, and Attention- Deficit/Hyperactivity Disorder (ADHD).

Our study’s third most important correlation was between Left-PrimMotor and the cerebellum. The motor cortex is also associated with alterations in ASD patients. Nebel et al.^144^ reported a delayed functional specialization within the motor cortex and alterations in both size and segregation of the primary motor cortex and that the functional sub-networks of the motor control system might be altered in autism. Mostofsky et al.^145^ observed a low motor ability in ASD related to increased white matter volume in the left hemisphere’s primary motor and premotor regions. We found a low correlation between Left-PrimMotor and the cerebellum for ASD, two important regions for motor control and skill, balance, and executive control of movements. Such a low correlation may cause problems in overall motor performance, thus interfering with socialization, which is commonly observed in ASD.

Regarding complex network measures, the most important measure for the model was the k-core, followed by the AEBC. K-Core decomposes the graph for finding important highly and mutually interconnected areas^146,147^. The k-core average was used for the calculation, which provides the degree of the subgraph in which all nodes have the same degree value, and helps identify small contiguous core areas in a network. High k-core values (pink dots) indicate its importance for the detection of TD, whereas low ones (blue dots) suggest ASD patients (Fig. 9, hence a weaker network connection among them. In contrast, EBC measures the average size of the largest community found by the edges betweenness method. For AEBC, low scores (blue dots) were important for detecting ASD, and high scores (pink dots) were important for detecting TD. Therefore, smaller communities can be detected by the presence of ASD. Higher values of efficiency were associated with TD patients and greater integration of networks and distributions of information in them. Therefore, the distribution of information in the functional networks of ASD patients is worse than that in TD. Concerning transitivity, a segregation network measure of the propensity of nodes to be grouped, higher values were associated with ASD, and low values indicated TD and the presence of more isolated communities clustered together.

The sliding process effectively differentiated TD from ASD patients since 30 patients achieved a 0.81 AUC and 0.81 mean accuracy. A statistical comparison between the sliding process and complete data showed no significant differences. Despite a lower performance with the use of the entire database, the technique could distinguish between ASD and TD patients with a significantly reduced amount of data, proving attractive for few data regarding ASD, as in^25,28^ (see Table 1). Furthermore, compared with some studies in Table 1^24–28^, our model, using these data augmentation techniques in a smaller amount of data, performed better in terms of AUC, accuracy, recall, and precision.

## Conclusions and future work

The workflow developed with the use of fMRI data could distinguish TD from ASD patients with both accuracy and AUC above 81%. The best pairwise statistical metric that captured brain changes due to ASD was SC, and the best-performing machine learning model was LG. According to the metric and the algorithm, the three most important brain connections with low values were established among Left-Sec Visual (visual cortex), Left-VentPostCing, and Left-PrimMotor with Outside defined BAS1 in ASD.

The functional connectivity of the Left-VentPostCing Posterior cingulate cortex is known to be reduced in ASD patients, which is consistent with our findings since this region is less connected to the cerebellum (Outside BAS1 region) in patients with ASD. Regarding complex networks, the brain networks of ASD patients showed more segregation, a weaker distribution of information across the network, and less connectivity. The sliding process employed effectively differentiated TD from ASD patients since a sample with 30 patients achieved 0.81 mean AUC and mean accuracy. A statistical comparison between the sliding process and complete data showed no significant differences. Therefore, the methodology is appropriate for cases of data of a small sample size.

Future studies may involve the application of the methodology to other fMRI data, as in^148^ for schizophrenia and fMRI data from ADHD-200 Global Competition. It can also be adopted with EEG data from patients with dystonia^149^. Other methodologies, such as the transfer learning method^150^, may be applied to small databases for comparison purposes.

### Supplementary Information

Supplementary Information.

## Data Availability

All data generated or analyzed during this study are included in this published article (and its Supplementary files).

## Grid search hyperparameter tuning

See Table 6.

**Table 6:** Hyperparameters for each classifier using Grid search optimizer.

Classifier	Hyperparameters and description	Values
RF	max_depth: Maximum depth of the tree	[1,2,5,10,20,80]
	max_features: Number of features to be considered toward a best split	[2, 3,5,10]
	min_samples_leaf : Minimum number of samples required to be at a leaf node	[1,2,3, 4, 5]
	min_samples_split: Minimum number of samples for the split of an internal node	[1,2,8, 10, 12,20]
	n_estimators: Number of trees in the forest	[1,2,3,5,10, 30,50,100, 200, 300,500]
SVM	Kernel: Specifies the kernel type to be used in the algorithm	[rbf, linear]
	Gamma: Kernel coefficient	[1e-3, 1e-4]
	C: Regularization parameter	[1, 10, 100, 1000]
NB	Var_smoothin:Portion of the largest variance of all features that is added to variances for calculation stability	range 1e-09 to 1
MLP	Activation: Activation function for the hidden layer	[identity, logistic, tanh, relu]
	Solver: Solver for weight optimization	[lbfgs, sgd, adam]
	Alpha: L2 penalty (regularization term) parameter	[0.0001,1e-5,0.01,0.001]
	Batch_size: Size of minibatches for stochastic optimizers	[1000,5000]
	Learning_rate: Learning rate schedule for weight updates	[constant, invscaling, adaptive]
	Learning_rate_init: The initial learning rate used	[0.001,0.01,0.1,0.2,0.3]
LR	C: Each value in Cs describes the inverse of r egularization strength	range 0.001 to 1000
	Penalty: Specifies the norm of the penalty	[l1, l2]

## Abbreviations

ABIDE: Brain Imaging Data Exchange
APL: Average shortest path length
ASD: Autism spectrum disorder
AUC: Area Under ROC Curve
BASC: Bootstrap Analysis of Stable Clusters
BC: Betweenness centrality
BM: Biweight Midcorrelation
BOLD: Blood Oxygenation Level Dependent
CC: Closeness centrality
DTI: Diffusion Tensor Imaging
EBC: Edge betweenness community detection
EC: Eigenvector centrality
ED: Entropy of the degree distributuion
EEG: Electroencephalogram
FC: Fastgreedy community detection
fMRI: Functional magnetic resonance imaging
GC: Granger Causality
GL: Graphical Lasso method
IC: Infomap community detection
Knn: Average degree of nearest neighbors
L-BFGS: Limited-memory Broyden Fletcher Goldfarb Shanno
LC: Leading eigenvector community detection
LG: Logistic regression
LPC: Label propagation community detection
LW: Ledoit-Wolf shrinkage
MC: Multilevel community detection
MI: Mutual Information
ML: Machine learning
MLP: Multilayer Perceptron
MSE: Mean Square Error
NB: Naive Bayes
PC: Pearson Correlation
PCP: Preprocessed Connectomes Project
RF: Random Forest
ROC: Receiver Operating Characteristic
ROI: Brain regions of interest
SC: Spearman Correlation
SCC: Canonical Correlation analysis
SHAP: SHapley Additive ExPlanations
SMD: Second moment of the degree distribution
SPC: Spinglass community detection
SVM: Support vector machines
TD: Typical development
TE: Transfer Entropy
Tuned CNN: Tuned convolution neural network
